# Arthroscopic coracoclavicular ligament reconstruction using allograft and TightRope in a patient with severe distal clavicle bone loss: A 5-year follow-up case report

**DOI:** 10.1097/MD.0000000000045623

**Published:** 2025-11-07

**Authors:** Kyeong Baek Kim, Sang-Min Lee, Suk Woong Kang

**Affiliations:** a Department of Orthopedic Surgery, Pusan National University Yangsan Hospital, Research Institute for Convergence of Biomedical Science and Technology, Yangsan, Republic of Korea; b Pusan National University School of Medicine, Yangsan, Republic of Korea; c Shiley Center for Orthopaedic Research and Education at Scripps Clinic, Department of Molecular Medicine, Scripps Research, LA Jolla, CA.

**Keywords:** acromioclavicular joint instability, allograft tendon, arthroscopic coracoclavicular ligament reconstruction, distal clavicle bone loss, TightRope system

## Abstract

**Rationale:**

Excessive distal clavicle resection can result in severe bone loss and disruption of the coracoclavicular (CC) ligament attachment, leading to significant vertical instability of the acromioclavicular joint. The optimal surgical approach in such cases remains controversial due to the technical challenges associated with achieving anatomical fixation.

**Patient concerns:**

A 40-year-old male construction worker presented with persistent shoulder pain, limited range of motion, and visible clavicular prominence 1 year and 7months after undergoing distal clavicle resection at an outside hospital following a traumatic fracture.

**Diagnoses:**

Radiography and physical examination revealed substantial bone loss in the distal clavicle and vertical instability, with a CC distance of 15.16 mm, confirming CC ligament insufficiency.

**Interventions:**

The patient underwent a single-tunnel arthroscopically assisted CC ligament reconstruction with allograft augmentation using the TightRope system and a peroneus brevis tendon allograft.

**Outcomes:**

At the 60-month follow-up, radiographs demonstrated maintained joint stability. The CC distance was reduced to 6.01 mm immediately postoperatively and was 9.30 mm at the final follow-up. Clinical scores significantly improved (Visual Analog Scale: 6→1, American Shoulder and Elbow Surgeons: 13→95, and University of California at Los Angeles: 9→35), and the patient returned to heavy manual labor 6 months postoperatively, maintaining full function.

**Lessons:**

In patients with severe distal clavicle bone loss, single-tunnel arthroscopically assisted CC ligament reconstruction with allograft augmentation can yield excellent long-term clinical and radiological outcomes, serving as a viable and effective treatment option.

## 1. Introduction

The acromioclavicular (AC) joint plays a vital role in connecting the upper limb to the axial skeleton, and its stability is maintained by the joint capsule and various ligamentous structures. The joint capsule and AC ligament contribute to horizontal stability, whereas the coracoclavicular (CC) ligament provides vertical stability.^[1,2]^ The CC ligament comprises the conoid and trapezoid ligaments, which differ both anatomically and functionally. The conoid ligament is located approximately 4.6 cm medial to the AC joint and primarily contributes to superior stability. In contrast, the trapezoid ligament, located approximately 2.5 cm medial and more anterior, resists anteroposterior (AP) compressive forces.^[3]^

Injuries to these structures following trauma can result in various forms of AC joint instability, for which the Tossy classification (3 types)^[4,5]^ and the extended Rockwood classification (6 types)^[5]^ are commonly used. In particular, surgical treatment may be required for complete dislocations classified as Rockwood Type III or higher, with the goal of anatomical reduction and functional stability.^[6]^

Historically, various surgical techniques such as pin fixation, hook plates, and wire fixation have been used; however, these techniques have limitations owing to complications and the potential need for revision surgery.^[7]^ Consequently, arthroscopically assisted anatomical reconstruction of the CC ligament has gained popularity, offering advantages such as minimal soft tissue damage, direct visualization of the pathology, and the ability to treat concomitant lesions.^[8–12]^

However, severe distal clavicle bone loss reduces residual clavicular strength and safe bone-bridge for tunnels, which increases fracture risk and compromises fixation biomechanics, as shown in cadaveric/biomechanical studies.^[7,13–16]^ In addition, in cases involving excessive distal clavicle resection, fixation to the original attachment site of the CC ligament becomes challenging because of bone loss, which makes vertical stability difficult to restore. Currently, no standardized surgical approach is available for such complex cases.

This case report describes a patient who developed significant bone deficiency and CC ligament instability following excessive distal clavicular resection at an external hospital. We performed single-tunnel arthroscopically assisted CC ligament reconstruction with allograft augmentation using the AC TightRope system (Arthrex Inc., Naples) and an peroneus allograft tendon and reported excellent clinical and radiographic outcomes over a 5-year follow-up period.

## 2. Case report

This study was approved by the institutional review board of Pusan National University Yangsan Hospital (IRB No. 55-2025-051). Written informed consent for publication was obtained from the patient. A 40-year-old male construction worker with no specific underlying disease, sustained a comminuted distal clavicle fracture after a motor vehicle accident. He subsequently underwent excessive distal clavicular resection at an outside hospital. Postoperatively, the patient experienced persistent shoulder pain, limited range of motion, and a visible clavicular prominence, which significantly interfered with daily and occupational activities. He presented to our outpatient clinic in 1 year and 7 months after the initial injury.

**Figure 1.**
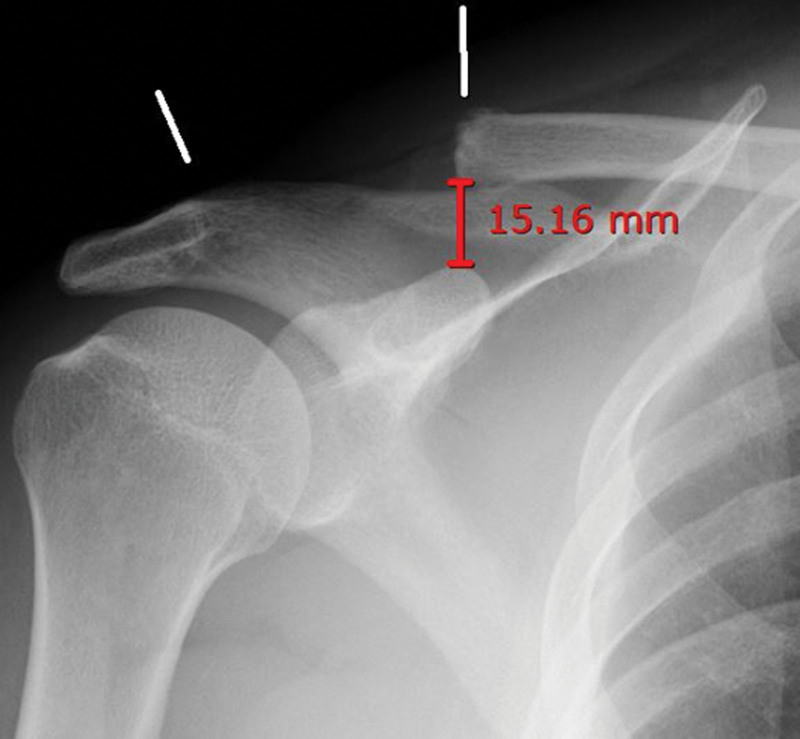
Preoperative anteroposterior radiograph of the right clavicle in a 40-year-old male patient presenting with persistent shoulder pain and dysfunction following excessive distal clavicle resection performed at an outside hospital. The white lines indicate the extent of the resected bone, with a coracoclavicular (CC) distance of 15.16 mm, suggesting significant vertical instability of the acromioclavicular joint.

Radiographs revealed an increased CC distance of 15.16 mm and extensive bone loss due to the prior resection (Fig. 1). Soft tissue protrusions have also been clinically observed. These findings indicated a loss of vertical stability due to the disruption of the CC ligament attachment site, which could not be adequately addressed using simple fixation.^[1,2]^

We planned arthroscopically assisted CC ligament reconstruction under general anesthesia, with the patient placed in the beach-chair position. Diagnostic arthroscopy was performed to evaluate intra-articular structures, including the rotator cuff and labrum. The distal clavicle stump and bony defects were confirmed. The anterior portal was established, and the coracoid was exposed between the superior and middle glenohumeral ligaments. Radiofrequency ablation was used to mark the graft attachment site while protecting the pectoralis minor and the brachial plexus.^[3,4]^

A longitudinal skin incision was made 3.5 cm medial to the AC joint along the Langer line. The distal clavicle was exposed after an incision of the deltotrapezial fascia.

We used a peroneus brevis tendon allograft and 2 cortical fixation buttons. The graft was prepared by whip-stitching each end for 30 mm with No. 0 FiberWire suture (Arthrex, Naples) and then folding it to create a double-strand construct with a final diameter of 4.5 to 5.0 mm.^[17]^ Due to the severe distal clavicle bone loss, creating a tunnel in the standard anatomical footprint was not feasible. Therefore, to minimize the risk of iatrogenic fracture, we planned the tunnel entry point at least 1 cm medial to the resected clavicular end. From this predetermined point, a 2.4-mm K-wire was drilled through the distal clavicle and into the posterocentral base of the coracoid under C-arm fluoroscopy. We utilized AP and Zanca views to confirm the pin’s trajectory and ensure it was well-centered in both bones. The tunnel was enlarged using a 3.0-mm cannulated drill.^[5]^

**Figure 2.**
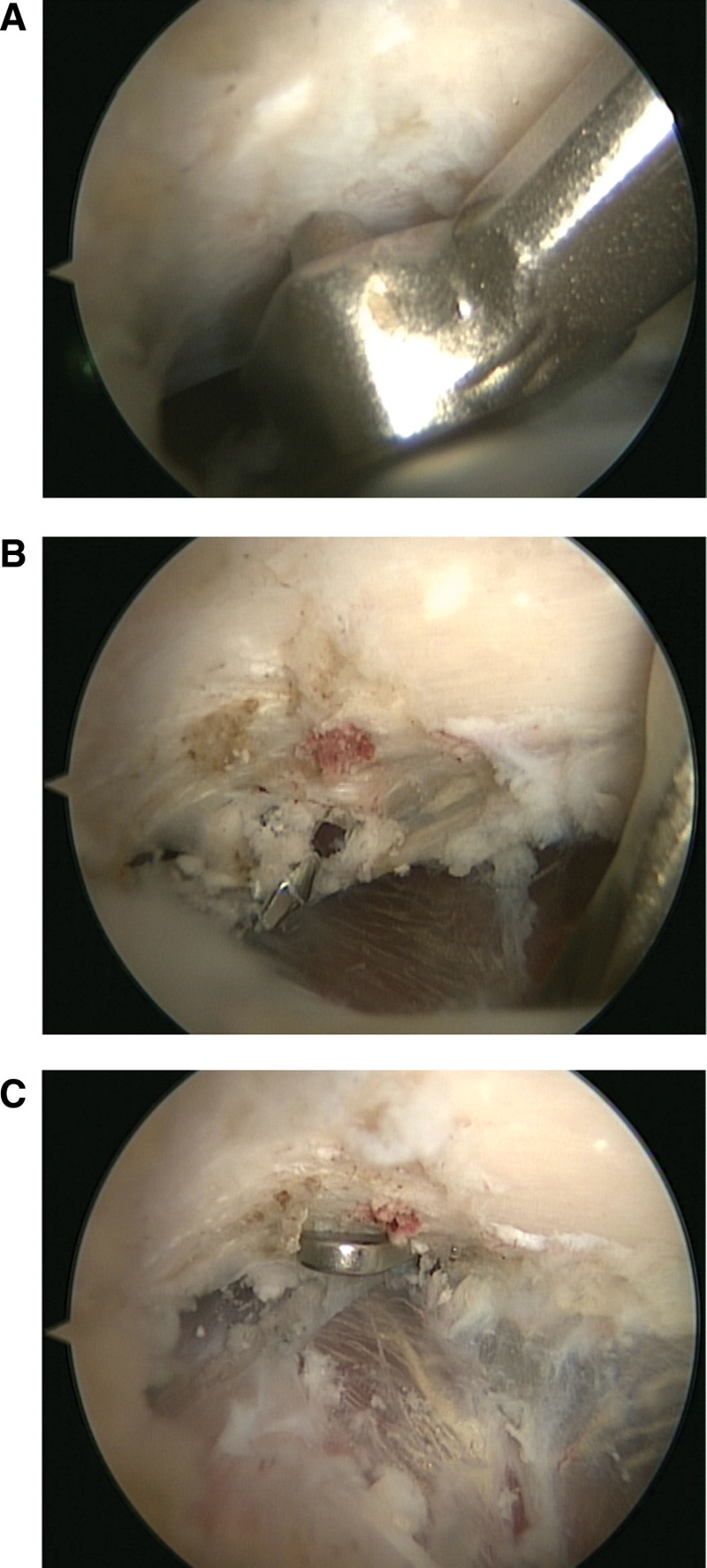
Arthroscopically assisted coracoclavicular ligament reconstruction. (A) A 2.4-mm K-wire is drilled from the planned point on the distal clavicle into the coracoid base. (B) The tunnel is enlarged with a 3.0-mm cannulated drill. (C) The allograft tendon is passed through the tunnel, and the TightRope system is secured to complete the fixation.

**Figure 3.**
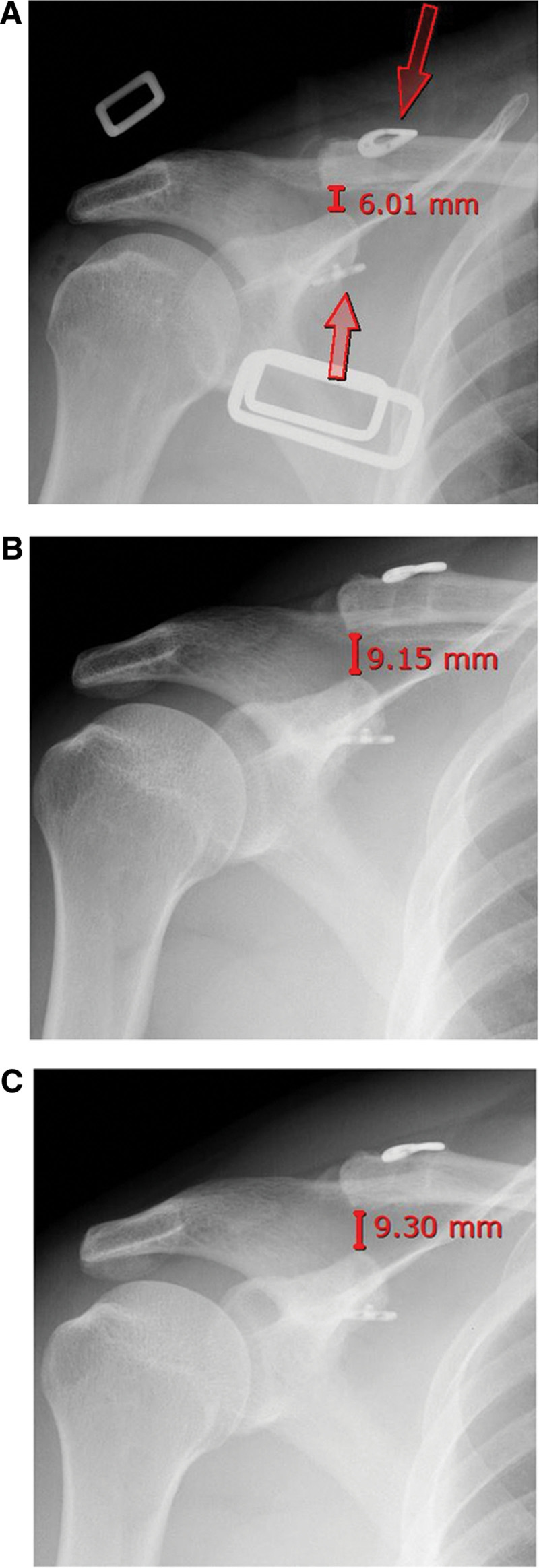
Postoperative and long-term follow-up radiographs. (A) An immediate postoperative anteroposterior radiograph shows anatomical reduction of the acromioclavicular joint, with a coracoclavicular (CC) distance of 6.01 mm. Red arrows indicate the 2 cortical fixation buttons. (B) At 3 years and (C) 5 years postoperatively, radiographs demonstrate that the joint remained stable, with CC distances of 9.15 and 9.30 mm, respectively. This long-term radiographic stability correlated with the patient’s successful return to his physically demanding occupation without functional limitations.

The guidewire was removed, and the allograft tendon along with the fixation button was delivered through the tunnel using passing sutures. The TightRope system was secured following anatomical reduction, and satisfactory reduction was confirmed using fluoroscopy (Fig. 2). This arthroscopic technique, using TightRope and allografts, provides the advantages of anatomic fixation and minimal soft tissue injury.^[7,8]^ Postoperative radiographs confirmed anatomic reduction, with a CC distance of 6.01 mm maintained after fixation (Fig. 3A).

Postoperatively, the patient wore a Kenny-Howard sling. Passive range of motion and pulley exercises (overhead shoulder movements assisted by a rope-and-pulley device, allowing the contralateral arm to mobilize the operated shoulder passively) were initiated at 6 weeks, followed by active motion and strengthening using a stick at 10 week Active and active-assist range-of-motion exercises are initiated at 8 to 10 weeks postoperatively. Strengthening begins at 10 to 12 weeks postoperatively. Staged rehabilitation plays a critical role in ligament and graft healing.^[9]^

At follow-up, radiographs demonstrated maintained reduction, and no distal clavicular instability was observed on physical examination. The CC distance was 9.15 mm at 36 months (Fig. 3B) and 9.30 mm at 60 months (Fig. 3C), which increased compared to the immediate postoperative value of 6.01 mm but remained within the normal range. Pain improved from Visual Analog Scale 6 to 1, American Shoulder and Elbow Surgeons score improved from 13 to 95, and University of California at Los Angeles score improved from 9 to 35.^[10]^ The patient returned to construction work 6 months postoperatively and remained functionally independent over 5 years.

## 3. Discussion

In the present study, we report a case of severe distal clavicle bone loss successfully managed with a single-tunnel CC reconstruction that achieved satisfactory clinical and radiographic outcomes. Despite the substantial deficiency in bone stock, this simplified technique provided stable fixation and functional recovery. Our results are comparable to those described by DeFoor et al,^[15]^ who treated distal clavicle insufficiency with an iliac crest autograft combined with AC–CC reconstruction and also reported favorable outcomes. By contrasting these approaches, our case highlights that even without bone graft augmentation, CC reconstruction can serve as a viable salvage option in select patients with severe bone loss.

The primary goal of AC joint injury treatment is to restore the anatomical and functional relationship between the distal clavicle and the scapula.^[1,2]^ This includes alignment on standard radiographs and restoration of AP stability on axillary views and functional kinetic linkage with the thorax and upper limb.^[3]^ Vertical stability depends on CC ligament reconstruction, whereas horizontal stability requires restoration of the AC ligament.

Traditional fixation methods, such as Kirschner wires, hook plates, and cerclage wires, have shown poor long-term outcomes owing to hardware-related complications and the need for removal.^[4]^ In particular, hook plates are associated with subacromial impingement and rotator cuff pathology and are unsuitable for long-term use.

The Weaver-Dunn procedure, which transposes the coracoacromial ligament to reconstruct the CC ligament, was introduced to overcome these limitations. However, this technique does not provide true anatomical restoration, and biomechanical studies have shown that it is weaker than the native CC ligament, with failure rates of up to 28%.^[5]^

More recently, arthroscopically assisted anatomical reconstruction has gained preference. It enables precise tunnel placement, minimal invasiveness, and concurrent treatment of other lesions such as rotator cuff tears or labral pathology.^[7,8,18,19]^ The combination of an allograft tendon and suspensory fixation systems such as TightRope provides both mechanical stability and biological healing potential.^[9,10,20,21]^

Severe distal clavicle bone loss compromises residual bone strength and limits the creation of a safe bone bridge for tunnels, thereby increasing the risk of fracture. In this context, high-strength tendon allografts (e.g., peroneus longus, semitendinosus) provide ultimate loads that exceed those of the native CC ligaments and can serve as the primary load-bearing structure when bone stock is deficient. Recent biomechanical investigations have demonstrated that suspensory or tunnel-sparing fixation using such grafts restores vertical stability while minimizing the risk of clavicular fracture in compromised bone conditions.^[22–24]^

However, this technique has some limitations. Tunnel malpositioning may lead to asymmetric loading, button cutout, or loosening.^[25,26]^ Additionally, suture-only fixation methods may lose up to 50% of their strength within 3 months, potentially leading to redislocation.^[27–29]^

In this case, the CC ligament insertion site was compromised due to severe bone loss following distal clavicle resection, making vertical vector restoration challenging. Although staged reconstruction with bone grafting and delayed ligament repair has been suggested,^[30]^ it carries risks, such as nonunion, delayed recovery, and structural instability. We achieved successful outcomes using one-stage arthroscopic TightRope and allograft reconstruction, leveraging the patient’s young age, soft tissue preservation, and accurate tunnel placement.

The patient maintained anatomical reduction and showed significantly improved American Shoulder and Elbow Surgeons and University of California at Los Angeles scores. He returned to heavy labor 6 months postoperatively and remained functionally stable for 60 months.^[31]^ This outcome reflected the combined benefits of technical precision and biological reconstruction.

We conclude that arthroscopically assisted CC ligament reconstruction using the TightRope and allografts is viable for treating AC joint instability with severe distal clavicle bone loss. Surgeons must be aware of potential complications such as fixation loss or malpositioned buttons. A technical pearl is the prevention of iatrogenic coracoid fracture during drilling. Given the small size of the coracoid process, precise guide pin placement is paramount. We emphasize the use of multiple fluoroscopic views, such as the AP and Zanca views, to confirm that the pin is perfectly centered in the coracoid base before enlarging the tunnel. Careful, slow drilling with a cannulated drill over the pin also minimizes stress on the bone.

This study has several limitations. First, as a case report, the findings are based on a single patient (n = 1) and cannot be generalized to all patients with similar conditions. Second, there was no control group for comparison with other surgical techniques. Finally, this report did not include a cost-effectiveness analysis comparing this procedure to alternative treatments, such as staged reconstruction with bone grafting. Future prospective studies with larger patient cohorts are needed to validate our findings.

## 4. Conclusions

Arthroscopically assisted CC ligament reconstruction using TightRope and allograft tendon provided excellent long-term radiographic and clinical outcomes in a patient with severe bone loss and AC joint instability following excessive distal clavicle resection.

## Author contributions

**Conceptualization:** Suk Woong Kang.

**Writing – original draft:** Kyeong Baek Kim, Sang-Min Lee.

**Writing – review & editing:** Kyeong Baek Kim, Sang-Min Lee, Suk Woong Kang.
